# Epstein–Barr virus infection is associated with the nuclear factor-kappa B p65 signaling pathway in renal cell carcinoma

**DOI:** 10.1186/s12894-022-00964-2

**Published:** 2022-02-07

**Authors:** Ali Farhadi, Sepide Namdari, Pei Pei Chong, Bita Geramizadeh, Abbas Behzad-Behbahani, Zamberi Sekawi, Sedigheh Sharifzadeh

**Affiliations:** 1 Diagnostic Laboratory Sciences and Technology Research Center, School of Paramedical Sciences, Shiraz University of Medical Sciences, Shiraz, Iran; 2 School of Biosciences, Taylor’s University, 47500 Subang Jaya, Selangor Malaysia; 3 Department of Pathology, Medical School of Shiraz University, Shiraz University of Medical Sciences, Shiraz, Iran; 4 Transplant Research Center, Shiraz University of Medical Sciences, Shiraz, Iran; 5 Department of Medical Microbiology and Parasitology, Faculty of Medicine and Health Sciences, Universiti Putra Malaysia, 43400 Serdang, Selangor Malaysia

**Keywords:** Epstein–Barr virus, Nuclear factor-kappa B, Renal cell carcinoma, p53, Ki-67, p16INK4a

## Abstract

**Background:**

There have been few studies regarding viral involvement in the pathogenesis of renal cell carcinoma (RCC). The aim of this study was to examine the possible association of Epstein–Barr virus (EBV) infection with clinicopathological features and cellular biomarkers including p53, p16INK4a, Ki-67 and nuclear factor-kappa B (NF-κB) in RCC tumors.

**Methods:**

In this prospective study, 122 histologically confirmed Formalin-fixed Paraffin-embedded RCC tissue specimens along with 96 specimens of their corresponding peritumoral tissues and 23 samples of blunt renal injuries were subjected to nested polymerase chain reaction (nPCR) in order to amplify EBV DNA sequences. The expression of p53, p16INK4a, Ki-67 and NF-κB was investigated by immunohistochemistry (IHC) assay. Statistical analysis was employed to demonstrate the possible associations.

**Results:**

Infection with EBV was found to be significantly associated with RCC. Our results indicate that p65 NF-κB signaling pathway is probably involved in EBV-mediated RCC pathogenesis. Moreover, we found p53, Ki-67 and cytoplasmic NF-κB expression to be associated with tumor nuclear grade in RCC patients. The expression of p53 and Ki-67 was associated with primary tumor category as well. In addition, p53 overexpression was significantly more frequent among nonconventional RCC tumors than the conventional histologic type.

**Conclusions:**

Infection with EBV is likely to play an important role in the development of RCC through the constitutive and permanent activation of NF-κB p65 signaling pathway. However, more experiments and supporting data are required to reach a decisive conclusion.

## Background

Renal cell carcinoma (RCC) is among the 10 most common cancers around the world and accounts for over 90% of renal neoplasms [1]. RCCs are a heterogeneous group of cancers originating from renal tubular epithelial cells [1, 2]. These tumors are often asymptomatic, have diverse clinical manifestations, and can be associated with hereditary syndromes [3]. Clear cell, papillary, and chromophobe RCCs account for the majority of RCC cases [4]. Recent advances in renal cell molecular biology and genetics have associated the disease with various hereditary and non-hereditary risk factors [3, 5]; however, they cannot explain all RCC cases. A growing body of evidence suggests that at least 15 percent of all human malignancies may be attributed to oncogenic viruses, one of which is Epstein–Barr virus (EBV) [6].

EBV, also known as human herpes virus 4, is a member of the subfamily Gammaherpesvirinae, genus Lymphocryptovirus which causes lifelong latent infections in memory B lymphocytes following primary infection [7]. Approximately 90% of the general population may be infected with this ubiquitous double stranded DNA virus as a result of a primary lytic infection in the oropharynx that can be asymptomatic or manifest as infectious mononucleosis [8]. EBV was present in the renal biopsies of individuals suffering from glomerular mesangial injury [9], and in the proximal tubule cells of human kidney tissue samples of patients with chronic interstitial nephritis [10]. In addition, the expression of EBV nuclear antigen 2 (EBNA2) in renal tubule cells has been reported to induce renal tumors in transgenic mice [11]. Furthermore, there are accumulating data available suggesting a role for EBV in RCC pathogenesis [12–14]. Together, these findings not only suggest that renal tissue is a likely reservoir of EBV, but also imply the oncogenic potential of EBV in the renal tissue. However, it is not yet known how EBV infection could be associated with RCCs.

p53 is a well-studied key tumor suppressor molecule that is mutated in half of human malignancies [15]. p53 expression has been reported to be an independent prognostic factor in patients with RCC in such a way that increased expression of p53 is associated with poor clinical outcome [16]. Furthermore, the overexpression of p53 has been shown to be an independent predictor of cancer-specific survival [17, 18], and correlate with high nuclear grade, tumor invasion, metastasis, late stage and cancer-caused death in clear cell RCC (ccRCC) [19]. In addition, a relationship between EBV infection and p53 expression has been reported in several cancers [20]. EBV nuclear antigen 3C (EBNA3C) has been reported to inhibit p53 binding to DNA, resulting in the repression of its transcriptional activity [21]. Further, it has been found that p53-mediated apoptosis is inhibited by latent membrane protein 1 (LMP1), another major protein of EBV [22].

p16INK4a is another tumor suppressor molecule which is involved in the retinoblastoma protein (Rb) pathway. It is well known that by arresting the S phase, p16INK4a contributes to the regulation of cell cycle progression [23]. The inactivation of *p16INK4a* gene by mutation, hypermethylation of the promoter, and homozygous deletion has been detected in various cancer cell lines including those obtained from RCC [24–26]. EBNA3C has been reported to inhibit p16INK4a to promote G1/S transition. Furthermore, EBV nuclear antigen 3A (EBNA3A) and EBNA3C have been shown to repress p16INK4A expression, leading to the proliferation of EBV-transformed cells [27].

Ki-67 is a nuclear protein which can be employed as a cell proliferation marker [28]. Ki-67 expression is known to correlate with ccRCC tumor grade and higher expression levels of this molecule is associated with worse prognosis [29]. This marker has also been considered a molecular representative of the aggressive behavior of tumor and response to therapy for survival outcome assessment in several tumors including RCC [30]. Furthermore, Ki-67 expression has been found to correlate with EBV infection in patients with nasopharyngeal carcinoma [31].

Nuclear factor-kappa B (NF-κB) is a pro-inflammatory transcription factor involved in a wide range of physiological and pathological cellular processes, including carcinogenesis [32]. The inactive form of NF-κB is localized in the cell cytoplasm while it is bound to the NF-κB inhibitor (IκB). During activation, IκB is degraded in the proteosome, leading to NF-κB release and translocation to the nucleus. While its activation in normal cells is short and transient, NF-κB becomes activated constitutively and permanently in tumor cells [33]. High rates of NF-κB activation have been associated with low apoptotic activity of tumor cells in ccRCC [34]. Further, higher expression of overall and nuclear NF-κB subunits has been reported to correlate with worse cancer-specific survival in RCC patients [35]. NF-κB signaling pathway has been found to be used by EBV to drive the cell cycle and lower the apoptotic activity of the cells. LMP1 of EBV interacts with NF-κB to promote G1/S transition, thereby giving the cells proliferation advantages [36].

In this study, we aimed to investigate the prevalence of EBV infection in RCC tumors. Further, due to the importance of p53, p16INK4a, Ki-67 and NF-κB in the viral oncogenesis, the expression of each biomarker was evaluated in all RCC tumor specimens and their corresponding peritumoral tissues as well. In addition, the correlation between EBV infection, demographic characteristics and the expression of cellular biomarkers were analyzed for the first time.

## Methods

### Study population

A total of 127 histologically confirmed RCC cases were included in this study, all of whom underwent surgery in Namazi hospital, Shiraz University of Medical Sciences, Iran. There were no restrictions on gender, age, ethnicity, or cancer stage at recruitment. The classification of different histological subtypes of RCC tumors was confirmed by two pathologists using hematoxylin and eosin (H&E) slides based on Heidelberg classification system [37]. Moreover, for 104 of these RCC cases, peritumoral kidney tissue sample was available. The histopathological grading of the nuclei of tumor cells was performed according to the four-group Fuhrman nuclear classification system [38]. The primary tumor staging was carried out according to the 2010 version of the American Joint Committee on Cancer (AJCC) TNM staging [39], and the Union for International Cancer Control (UICC) classification system [40]. Furthermore, 23 samples of normal kidney tissue from patients with blunt renal injuries were available for this study.

### Sample preparation and DNA extraction

Four 5 µm thick slices were cut and collected in autoclaved Eppendorf microcentrifuge tubes (1.5 ml) for each patient. In order to avoid contamination among specimens, precautions were taken including cutting one case at a time, changing the microtome blade before cutting every new case and cleaning the microtome and the workspace thoroughly with ethanol between each case. Moreover, paraffin blocks without tissue were sectioned after cutting every real specimen and included as negative controls of DNA extraction procedures. Genomic DNA was extracted from the Formalin-fixed Paraffin-embedded tissue sections using QIAamp DNA FFPE Tissue Kit (Qiagen, Hilden, Germany) according to the manufacturer’s protocol. Extracted DNA was eluted in 50 ml ATE buffer and stored at −70 °C until analyzed. The total amount of DNA was quantified by a NanoDrop (ND-1000) spectrophotometer (peQLab Biotechnologie, Erlangen, Germany). The quality of the extracted DNA was evaluated by PCR amplification of each sample using ß-globin gene specific primers, PC03 and PC04 producing a 110 bp PCR product as described by Saiki et al. [41]. Only positive samples were chosen for further analysis.

### Nested PCR amplification of EBV DNA

A nested PCR system for diagnosis of EBV infections in tissue specimens from patients with RCC was applied as described by Espy et al. [42] using oligonucleotide primers directed to a conserved region of EBV genome encoding the capsid protein gp220 (BamHI region) (Table 1). All primers were custom synthesized by Bioneer (Daejeon, Korea). EBV positive specimens were amplified and analyzed in duplicate in order to ensure consistency and reliability of the results. Sterile distilled water was used as negative control for all reactions.

**Table 1:** Primer names and sequences

Primer name	Sequences (5′–3′)	Product size (bp)	References
EBV-1F-outer	GTGCCTCGTCTACCTCTGTTC	277	Espy et al. [42]
EBV-2R-outer	TTGATTCTCGTGGTCGTGTTCC		
EBV-3F-inner	CAGTGCCTCCGCCTGAGCCGCT	170	
EBV-4R-inner	GGTCAGATTTTGCAATATATTTT		
PC03	ACACAACTGTGTTCACTAGC	110	Saiki et al. [41]
PC04	CAACTTCATCCACGTTCACC		

### Sensitivity assay and control cell line

In order to test the sensitivity of primers used for nested PCR amplification of EBV DNA, LCL-PI7 (Pasteur Institute of Iran, Tehran), an EBV transformed lymphoblastoid cell line, with approximately 50 copies of the EBV genome integrated in each cell was employed. Ten-fold dilutions of EBV DNA-containing LCL-PI7 cells were made. The dilution series started with 10^5^ cells/ml (corresponding to approximately 5 × 10^6^ of EBV DNA copies) and ended with 1 cell/ ml of LCL-PI7 cells (corresponding to approximately 50 viral target copies). Genomic DNA was extracted from each of these dilutions and tested using nested PCR assay as described for the sample group.

### Immunohistochemical staining

Paraffin-embedded tissue samples were sectioned at 5 µm thickness and mounted onto slides pre-coated with 300 ml of poly-l-lysine solution (0.1% w/v, Sigma–Aldrich; St. Louis, MO). Deparaffinization was performed by fresh xylene, followed by endogenous peroxidase activity quenching by 10% H_2_O_2_ solution. Epitope retrieval was carried out by heat-induced microwave treatment in the corresponding buffer solutions (Table 2). Protein block agent (Dako, Glostrup, Denmark) was used in order to block non-specific binding sites. After incubation with antibodies and buffer washes, sections were covered with EnVision + Dual Link System-HRP solution (Dako) for 30 min at room temperature. The reaction was visualized using 3,3’-diaminobenzidine (DAB) staining. Sections without primary antibody served as negative controls in each run. Positive controls were included as presented in Table 2.

**Table 2:** Antibodies used for immunohistochemistry assay

Antibody	Source	Clone and manufacturer	Antigen retrieval solution pH	Dilution and incubation	Positive control
Anti-p53	Mouse monoclonal IgG1	DO-7, Dako, Glostrup, Denmark	10 mM Citrate buffer pH 6.0	1:50, overnight at 4 °C	Breast cancer sections with p53-positive nuclear staining
Anti-p16INK4a	Mouse monoclonal IgG1	JC8, Santa Cruz Biotechnology, California, USA	10 mM Tris–EDTA buffer pH 9.0	1:50, overnight at 4 °C	Formalin-fixed HeLa cell blocks
Anti-Ki-67	Mouse monoclonal IgG1	MIB-1, Dako, Glostrup, Denmark	10 mM Citrate buffer pH 6.0	1:100, overnight at 4 °C	Intestine tissue sections
Anti-NF-κB (p65)	Mouse monoclonal IgG1	Santa Cruz Biotechnology, California, USA	10 mM Citrate buffer pH 6.0	1:400, 1 h at room temperature	Formalin-fixed HeLa cell blocks

### Evaluation of immunohistochemical staining

Immunostainings with p16INK4a, p53, Ki-67 and NF-κB antibodies were examined in a double-blind protocol and scored under 400 × magnification standard light microscopy. In the case of p53 and Ki-67 immunoexpression, only nuclear staining was interpreted positive and the evaluation of results was performed according to a semi-quantitative scoring system adopted from a previous study [43] (Table 3). The 10% cut-off value was selected for the interpretation of p53 and Ki-67 nuclear immunostaining results, so that the reactions were considered positive while 10% or more of the cancer cells were stained [44, 45].

**Table 3:** Semi-quantitative scoring system of p53 and Ki-67 immunostaining

Percentage of immunoreactive cells	Score	p53 and Ki-67 protein overexpression assessment	Reference
No reactivity	0	Negative	Zigeuner et al. [43]
Less than 10%	1	Negative	
10–25%	2	Positive	
26–50%	3	Positive	
51–75%	4	Positive	
76–90%	5	Positive	
More than 90%	6	Positive	

For p16INK4a immunostaining analysis, the percentages of tumor cells reactive with p16INK4a antibody were evaluated using a semi-quantitative scoring system adopted from previous studies [46, 47] (Table 4). Both nuclear and cytoplasmic staining were interpreted as positive reaction. Samples were counted as positive when more than 5% of cells (cut-off) were stained.

**Table 4:** Semi-quantitative scoring system of p16INK4a immunostaining

Percentage of immunoreactive cells	Staining pattern	p16INK4a expression assessment	Reference
0–5%	Negative	Negative	Gabrielli Fregonesi et al. [46]
6–10%	Sporadic	Positive (low expression)	
11–30%	Focal	Positive (moderate expression)	
More than 30%	Diffuse	Positive (high expression)	

The evaluation of NF-κB immunostaining results was performed based on the staining intensity as well as the percentage of positive cells, according to Zhou et al. [48] (Table 5). The nuclear expression of NF-κB p65 was examined separately to investigate the NF-κB/relA signaling pathway which can be interpreted through p65 translocation from cytoplasm to the nucleus [49].

**Table 5:** Semi-quantitative scoring system of NF-κB immunostaining

Percentage of immunoreactive cells	Percentage-based score	Staining intensity	Intensity-based score	Reference
0–5%	0	No staining	0	Zhou et al. [48]
6–25%	1	Straw yellow	1	
26–50%	2	Brown	2	
51–75%	3	Tan	3	
More than 75%	4			

For statistical analysis, the scoring index was calculated by addition of percentage-based scores and intensity-based scores, and the cut-off value was selected as three so that < 3 was defined as low expression and ≥ 3 was considered as high expression

### Statistical analysis

Statistical analysis was performed using SPSS version 25.0 (SPSS Institute, Chicago, IL, USA). The associations between clinicopathological characteristics and the presence of EBV and/or the expression of cellular biomarkers were analyzed using two-sided Chi-square or Fisher’s exact test, where appropriate. To assess the significance of the difference between the prevalence of viral DNA in tumor tissue specimens and the corresponding peritumoral tissues, McNemar test was used. However, Chi-square/ Fisher’s exact test was applied to evaluate the significance of the difference between the prevalence of viral DNA in RCC tissue specimens and normal kidney tissue blocks. *P* values less than 0.05 were considered to be statistically significant.

## Results

### Study set

Tissue blocks of a total number of 122 patients with renal cell carcinoma (mean age: 54 years; range: 3–81) and 96 specimens of their corresponding surrounding normal kidney tissue in addition to 19 tissue blocks from patients with renal trauma (12 men and 7 women; mean age: 39 years) were available for analysis. DNA fragments of ß-globin gene were not amplifiable in tumoral tissue blocks of five patients, peritumoral tissue blocks of eight patients, and four specimens from renal trauma specimens, all of which were excluded from the study. The clinicopathological features of the patients are presented in Table 6.

**Table 6:** The comparison of clinicopathological characteristics between EBV positive and negative RCC patients (n = 122)

Variable	All patients	EBV positive patients N (%)	EBV negative patients N (%)	*p* value
Total	122 (100)	33 (27.0)	89 (72.9)	
Gender				
Male	70 (57.4)	17 (51.1)	53 (59.5)	0.425
Female	52 (42.6)	16 (48.4)	36 (40.4)	
Age				
≥ 54	58 (47.5)	17 (51.1)	41 (46)	0.592
˂ 54	64 (52.5)	16 (48.4)	48 (53.9)	
Tumor location				
Right	67 (54.9)	19 (57.5)	48 (53.9)	0.719
Left	55 (45.1)	14 (42.4)	41 (46)	
Tumor grade				
G1	55 (45.1)	13 (39.3)	42 (47.1)	0.893
G2	44 (36.1)	13 (39.3)	31 (34.8)	
G3	20 (16.4)	6 (18.1)	14 (15.7)	
G4	3 (2.4)	1 (3)	2 (2.2)	
pT category				
pT1	58 (47.5)	16 (48.4)	42 (47.1)	0.725
pT2	43 (35.2)	13 (39.3)	30 (33.7)	
pT3	19 (15.6)	4 (12.1)	15 (16.8)	
pT4	2 (1.6)	0	2 (2.2)	
Histologic types				
Conventional	77 (63.1)	23 (69.7)	54 (60.6)	0.357
Papillary	26 (21.3)	6 (18.1)	20 (22.4)	
Chromophobe	14 (11.5)	2 (6)	12 (13.4)	
Collecting duct	1 (0.8)	1 (3)	0	
Unclassified	4 (3.3)	1 (3)	3 (3.3)	

*pT* primary tumor

### Detection of EBV

It appeared that the sensitivity of EBV1F/EBV2R primers at the primary PCR was approximately 5 × 10^3^ viral target copies/ml. However, the results from the secondary PCR amplification showed the ability of EBV3F/EBV4R primers to detect less than 500 viral genome copies/ml. Of 122 RCC specimens, 96 blocks of their corresponding surrounding tissue and 19 tissue blocks from patients with renal trauma, 33 (27%), 8 (8.3%) and 1 (5.2%) tested positive for EBV DNA, respectively. EBV DNA was not detectable in two RCC cases whereas their related peritumoral tissues tested positive for EBV DNA. The comparison of the presence of EBV DNA between RCC tumor tissues and their corresponding peritumoral tissues showed a statistically significant difference (*p* < 0.001, McNemar Test). Furthermore, when RCC tumor tissues were compared with tissue blocks from patients with renal trauma in terms of the presence of EBV DNA, a significant difference was found (*p* = 0.044, Fisher’s Exact Test).

### Immunohistochemical staining of p53, p16INK4a, Ki-67 and NF-κB proteins

**Fig. 1:**
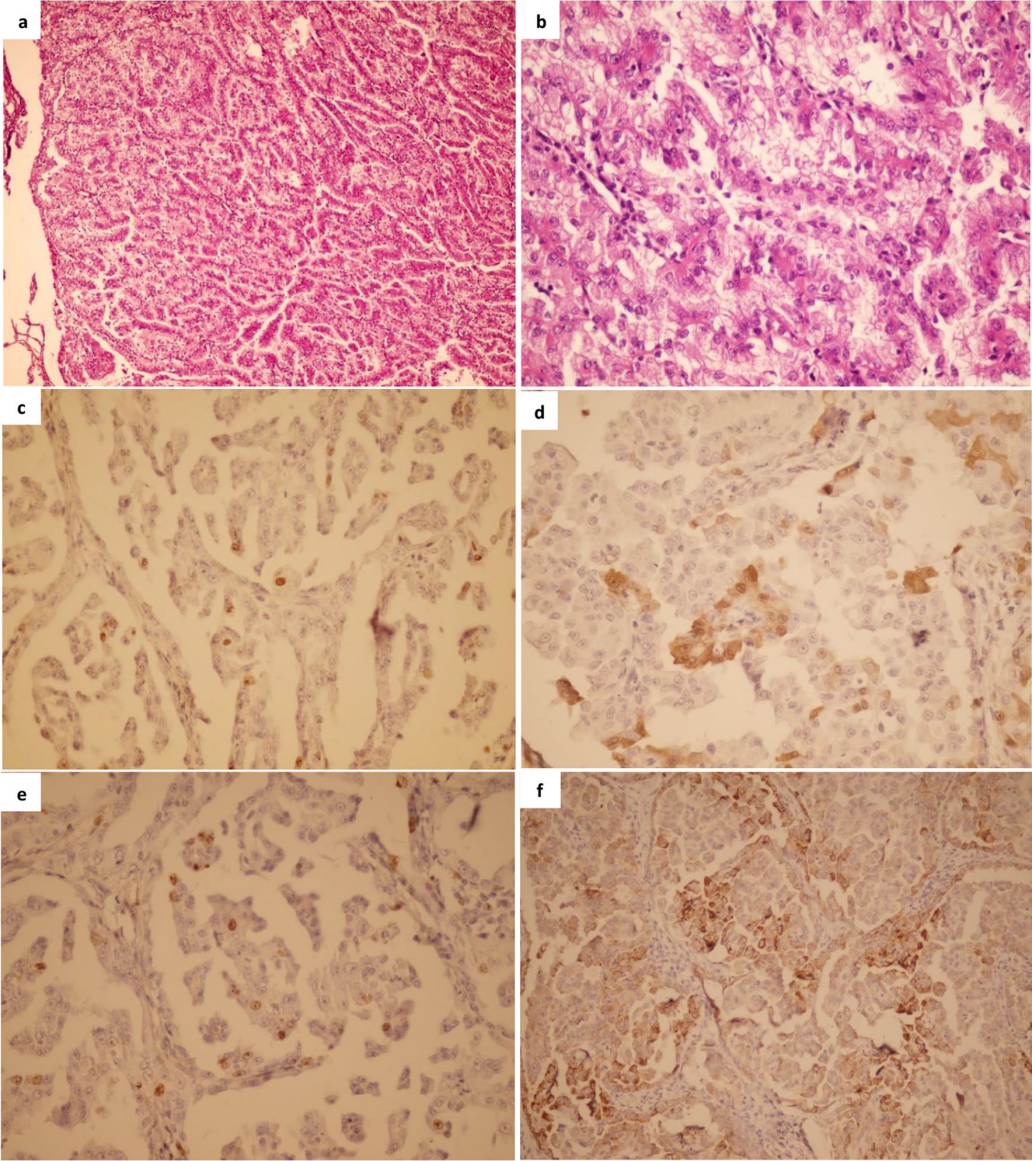
Representative hematoxylin and eosin (H&E) staining and immunohistochemical analysis of cellular biomarkers of tissue specimens from a patient with type II papillary RCC with Fuhrman nuclear grade 3. **a** hematoxylin staining, 100× **b** hematoxylin staining, 400× **c** p53 immunostaining, 400× **d** p16INK4a immunostaining, 400× **e** Ki-67 immunostaining, 400× **f** NF-κB (p65) immunostaining, 400×

The immunohistochemical staining of p53, Ki-67, p16INK4a and NF-κB was carried out for 118 RCC cases. The immunoreactivity of p53 was detected in 27 (22.9%) of all RCC cases. Semi-quantitative analysis showed immunoreactivity of less than 10% of tumor cells in 16 (59.3%) cases, 10% to 25% in 9 (33.3%) cases, and 26% to 50% in 2 (7.4%) cases of p53 positive RCC tumors. Since the overexpression of p53 was defined as nuclear immunostaining in ≥ 10% of tumor nuclei, only 11 (9.3%) of total RCC tumors were considered cases of p53 overexpression. Moreover, the association between the detection of p53 protein overexpression and clinicopathological parameters in RCC patients was examined (Table 7). It was revealed that p53 positivity was significantly associated with Fuhrman nuclear grade (*p *= 0.006). Furthermore, p53 overexpression was found to be significantly more frequent among patients with nonconventional RCCs than those with conventional type (*p* = 0.020). Example of p53 protein expression in a patient with type II papillary RCC is demonstrated in Fig. 1.

**Table 7:** The comparison of clinicopathological characteristics between p16INK4a/Ki-67/ p53 positive and negative RCC patients (n = 118)

Variable	p53 Pos N (%)	p53 Neg N (%)	*p* value	P16 Pos N (%)	P16 Neg N (%)	p value	Ki-67 Pos N (%)	Ki-67 Neg N (%)	*p* value	High cyto- NF-κB N (%)	Low cyto- NF-κB N (%)	p value	High nuclear NF-κB N (%)	Low nuclear NF-κB N (%)	*p* value
Total	11 (9.3)	107 (90.7)		24 (20.3)	94 (79.7)		24 (20.3)	94 (79.7)		98 (83.0)	20 (16.9)		70 (59.3)	48 (40.7)	
Gender															
Male	7 (63.6)	61 (57)	0.757	17 (70.8)	51 (54.3)	0.169	18 (75)	50 (53.2)	0.065	56 (57.1)	12 (60.0)	> 0.999	38 (54.3)	30 (62.5)	0.449
Female	4 (36.4)	46 (43)		7 (29.1)	43 (45.7)		6 (25)	44 (46.8)		42 (42.9)	8 (40.0)		32 (45.7)	18 (37.5)	
Age															
≥ 54	6 (54.5)	57 (53.3)	> 0.999	10 (41.6)	53 (56.4)	0.253	15 (62.5)	48 (51.1)	0.364	52 (53.1)	11 (55)	> 0.999	42 (60)	21 (43.7)	0.094
˂ 54	5 (45.5)	50 (46.7)		14 (58.3)	41 (43.6)		9 (37.5)	46 (48.9)		46 (46.9)	9 (45)		28 (40)	27 (56.2)	
Tumor location															
Right	7 (63.6)	58 (54.2)	0.752	14 (58.3)	51 (54.3)	0.819	11 (45.8)	54 (57.4)	0.361	56 (57.1)	9 (45.0)	0.336	36 (51.4)	29 (60.4)	0.353
Left	4 (36.4)	49 (45.8)		10 (41.6)	43 (45.7)		13 (54.2)	40 (42.6)		42 (42.9)	11 (55.0)		34 (48.6)	19 (39.6)	
Tumor grade															
G1	3 (27.2)	50 (46.7)	0.006	9 (37.5)	44 (46.8)	0.280	3 (12.5)	50 (53.2)	< 0.0001	36 (36.7)	17 (85.0)	0.003	30 (42.9)	23 (47.9)	0.485
G2	2 (18.1)	40 (37.3)		8 (33.3)	34 (36.1)		7 (29.2)	35 (37.2)		39 (39.8)	3 (15.0)		23 (32.8)	19 (39.6)	
G3	6 (54.5)	14 (13.1)		7 (29.1)	13 (13.8)		13 (54.2)	7 (7.4)		20 (20.4)	0		15 (21.4)	5 (10.4)	
G4	0	3 (2.8)		0	3 (3.2)		1 (4.1)	2 (2.1)		3 (3.1)	0		2 (2.9)	1 (2.1)	
G1 + G2	5 (45.5)	90 (84.1)	0.007	17 (70.8)	78 (82.9)	0.246	10 (41.7)	85 (90.4)	< 0.0001	75 (76.5)	20 (100)	0.012	53 (75.7)	42 (87.5)	0.156
G3 + G4	6 (54.5)	17 (15.9)		7 (29.1)	16 (17.0)		14 (58.3)	9 (9.6)		23 (23.50	0		17 (24.3)	6 (12.5)	
pT category															
pT1	6 (54.5)	49 (45.8)	0.014	11 (45.8)	44 (46.8)	0.209	8 (33.3)	47 (50)	0.0002	42 (42.8)	13 (65)	0.254	31 (44.3)	24 (50)	0.364
pT2	3 (27.2)	40 (37.3)		6 (25.0)	37 (39.4)		5 (20.8)	38 (40.4)		37 (37.7)	6 (30)		29 (41.4)	14 (29.2)	
pT3	1 (9.1)	18 (16.8)		7 (29.1)	12 (12.7)		10 (41.7)	9 (9.6)		18 (18.4)	1 (5)		10 (14.3)	9 (18.7)	
pT4	1 (9.1)	0		0	1 (1.1)		1 (4.2)	0		1 (1.0)	0		0	1 (2.1)	
pT1 + pT2	9 (81.9)	89 (83.2)	> 0.999	17 (70.8)	81 (86.1)	0.122	13 (54.2)	85 (90.4)	0.0001	79 (80.6)	19 (95)	0.190	60 (85.7)	38 (79.2)	0.351
pT3 + pT4	2 (18.1)	18 (16.8)		7 (29.1)	13 (13.8)		11 (45.8)	9 (9.6)		19 (19.4)	1 (5)		10 (14.3)	10 (20.8)	
Histologic types															
Conventional	3 (27.3)	70 (65.4)	0.020 ^a^	12 (50)	61 (64.9)	0.239	12 (50)	61 (64.9)	0.239 ^a^	61 (62.2)	12 (60)	0.850	41 (58.6)	32 (66.6)	0.373
Nonconventional	8 (72.7)	37 (34.6)		12 (50)	33 (35.1)		12 (50)	33 (35.1)		37 (37.7)	8 (40)		29 (41.4)	16 (33.3)	
Papillary	6 (54.5)	20 (18.7)		9 (37.5)	17 (18.1)		8 (33.3)	18 (19.1)		23 (23.4)	3 (15)		20 (28.6)	6 (12.5)	
Chromophobe	1 (9.1)	13 (12.1)		1 (4.1)	13 (13.8)		0	14 (14.9)		9 (9.2)	5 (25)		5 (7.1)	9 (18.7)	
Collecting duct	0	1 (0.9)		0	1 (1.1)		1 (4.2)	0		1 (1.0)	0		1 (1.4)	0	
Unclassified	1 (9.1)	3 (2.8)		2 (8.3)	2 (2.1)		3 (12.5)	1 (1.1)		4 (4.1)	0		3 (4.3)	1 (2.1)	
EBV															
Positive	5 (45.4)	28 (26.2)	0.287	8 (33.3)	25 (26.6)	0.611	8 (33.3)	25 (26.6)	0.611	31 (31.6)	2 (10)	0.058	29 (41.4)	4 (8.3)	< 0.0001
Negative	6 (54.5)	79 (73.8)		16 (66.6)	69 (73.4)		16 (66.6)	69 (73.6)		67 (68.4)	18 (90)		41 (58.6)	44 (91.7)	

*Pos* positive, *Neg* negative, *Cyto* cytoplasmic, *P16* p16INK4a, *pT* primary tumor

The expression of p16INK4a was observed in 24 (20.3%) cases. Specifically, 94 (79.7%) cases were negative, 7 (5.9%) cases showed low expression, 15 (12.7%) cases exhibited moderate and 2 (1.7%) cases demonstrated high expression of p16INK4a. Immunostaining of p16INK4a was predominantly detected in the tumor cell nuclei and in some cases accompanied by additional staining in the cell cytoplasm. Strong antibody localization in the cytoplasm was, nevertheless, detected in three RCC cases. Investigating the expression of p16INK4a in relation to that of p53 in 118 cases of RCC, a significant association was found (*p* = 0.045).

Immunostaining for Ki-67 was localized to the nuclei of the cancer cells with a fine brown granularity. The representative positive staining of Ki-67 in a tissue sample from a patient with type II papillary RCC is demonstrated in Fig. 1. The evaluation of Ki-67 immunostaining showed a wide range of indices, from 1 to 60%, with a mean value corresponding to 9.1%. Using nuclear immunostaining of Ki-67 in ≥ 10% of tumor nuclei as cut-off, the overexpression of Ki-67 protein was considered positive in 24 (20.3%) out of 118 RCC cases. There was a significant association between Ki-67 positivity and Fuhrman nuclear grade (*p* < 0.0001). Moreover, we found a significant association between Ki-67 overexpression and primary tumor categories (*p* = 0.0002). Ki-67 overexpression was investigated in relation to p53 and p16INK4a protein expression in 118 RCC cases as well (Table 8). Data analysis showed a significant association between Ki-67 and p53 (*p* = 0.045), as well as between Ki-67 and p16INK4a expression in RCC tumors (*p* = 0.043).

**Table 8:** The association between different biomarkers among RCC patients

		p16INK4a		*p* value	Ki-67		*p* value	NF-κB cytoplasmic		*p* value	NF-κB nuclear		*p* value
		Positive	Negative		Positive	Negative		High	Low		High	Low	
p53	Positive	5	6	0.045	5	6	0.045	11	0	0.207	10	1	0.027
	Negative	19	88		19	88		87	20		60	47	
p16INK4a	Positive				9	15	0.043	20	4	> 0.999	7	17	0.001
	Negative				15	79		78	16		63	31	
Ki-67	Positive							24	0	0.012	14	10	> 0.999
	Negative							74	20		56	38	
NF-κB cytoplasmic	High										66	52	0.0001
	Low										4	16	

High cytoplasmic NF-κB expression was detected in 98 (83.0%) cases whereas high nuclear NF-κB expression was observed in 70 (59.3%) RCC cases. Cytoplasmic NF-κB expression was found to be significantly associated with Fuhrman nuclear grade (*p* = 0.003). Furthermore, it was revealed that high nuclear NF-κB expression was significantly associated with EBV infection in RCC cases (*p* < 0.0001). Examining the relationship between the expression of NF-κB and that of p53, Ki-67 and p16INK4a, several associations were discovered (Table 8); cytoplasmic NF-κB expression was associated with Ki-67 overexpression (*p* = 0.012); nuclear NF-κB expression was associated with p53 overexpression (*p* = 0.027); and there was a negative association between nuclear NF-κB expression and p16INK4a expression in RCC cases (*p* = 0.001).

## Discussion

In spite of the increasing amount of evidence for the impact of viral infections on cancer development, limited data exists on the relationship between viral infections and RCC. Taking into account that renal tissue is a probable reservoir of EBV [50], and considering the oncogenic potential of this virus [11, 51], we conducted this study to investigate the possible role of EBV in RCCs. We studied 122 RCC cases along with 96 corresponding normal kidney tissue samples and 19 tissue specimens from patients with renal trauma. Our results suggest a correlation between EBV infection and RCC. This finding is in line with a study by Shimakage et al. on 9 RCC cases and 2 RCC cell lines, all of which were found to be infected with EBV [12]. The frequency of EBV infection in RCC tumors was reported to be 15.6% and 29.6% in two other former studies [13, 14]. Our results, along with the others’, show that EBV infection is common in RCCs. Further, in spite of a previous study reporting a correlation between EBV infection and tumor grade in RCC patients [14], we did not find such a relationship. This may have been due to the small sample size of the mentioned study which consisted of 27 RCC patients and also missing data on tumor grades in 2 RCC cases.

p53 is a tumor suppressor with a high rate of mutations in human tumors [52]. In surgical pathologic evaluation, immunohistochemistry is known as a well-established means to evaluate p53 status, and positive staining is linked to the accumulation of mutant p53 protein [43]. The current study revealed that elevated levels of p53 expression were significantly more common in nonconventional RCC histologic subtypes compared to the conventional subtype. This finding is in agreement with a previous study which demonstrated p53 overexpression in 70%, 27.3%, and 11.9% of papillary, chromophobe, and conventional subtypes of RCC, respectively [43]. Furthermore, the fact that the overexpression of p53 protein was accompanied by higher grades of the tumor in our study, which is in agreement with other studies [53], suggests a straightforward link between p53 expression and tumor progression in RCCs. However, our study revealed no relationship between p53 expression and EBV infection in these cases.

p16INK4a protein which inhibits the progression of cell cycle is shown to be downregulated in several cancer cell lines including those derived from RCC tumors [24, 25]. The complete or approximately complete loss of p16INK4a has been frequently reported in RCC specimens, strongly suggesting that low p16INK4a levels participate in the pathogenesis of RCC [54, 55]. In the present study, the expression of p16INK4a was absent in the majority (79.7%) of RCC cases which is consistent with the referred previous studies. Furthermore, the investigation of p16INK4a expression in relation to EBV infection in RCC cases revealed no correlation.

Another potential candidate to determine the prognosis and survival in RCC cases is Ki-67 biomarker. Ki-67 is regarded as an index of the tumor proliferative activity [56]. The findings of this study confirm those of previous reports that high-level expression of Ki-67 was strongly associated with high primary tumor stage [56–58]. In RCCs, high-level staining of Ki-67 has been found to correlate with high tumor grade and poor prognosis [29, 59]. Our results confirm increased expression of Ki-67 in RCC tumors of higher Fuhrman grades and yet demonstrate no association between Ki-67 expression and EBV infection in RCC.

NF-κB dysregulation, among others, is a pivotal step in the occurrence and development of many tumors. The expression of NF-κB is upregulated in several cancers, including head and neck squamous cell carcinoma (HNSCC), pancreatic ductal adenocarcinoma, colorectal cancer, and gastric cancer [60, 61]. The expression and activation of p65, the most well-studied NF-kB subunit in cancer, is increased in RCC tissues [62]. p65 has been correlated with apoptosis as well as proliferation markers in RCC [19]. The activation of NF-κB induces anti-apoptotic factors such as the anti-apoptotic Bcl-2 family of proteins [63]. Moreover, the expression of NF-κB p65 has been associated with that of vascular endothelial growth factor (VEGF) in ccRCC [64]. Hence NF-κB is probably involved in the development and progression of RCC. In this study, cytoplasmic NF-κB was found to be associated with RCC tumor grade in such a way that high tumor nuclear grades were accompanied by high levels of cytoplasmic NF-κB. Moreover, we found high nuclear NF- κB expression to be associated with EBV infection in RCC cases.

Epstein–Barr virus plays a key role in driving the cell cycle and oncogenesis of EBV-positive neoplasms. Multiple genes and signaling pathways are involved in the occurrence of EBV-related neoplasms, including the interaction of viral and host genes [36]. EBV-encoded genes activate oncogenes such as Bcl-2 and MYC and inhibit tumor suppressor genes such as p53, p16INK4A, PTEN, etc. [36]. Further, the latent proteins and miRNAs of EBV drive the cell cycle into various signaling pathways such as NF-κB, phosphoinositide-3-kinase/protein kinase B (PI3K/AKT), mitogen-activated protein kinase (MAPK), Janus kinase/signal transducer and activator of transcription (JAK/STAT), Wnt/β-catenin, and transforming growth factor-β (TGF-β) [60]. There is evidence that NF-κB activation is enhanced by LMP1, the most studied EBV oncoprotein, leading to increased cell proliferation, migration and invasion [36]. Further, it has been found that the combination of LMP1 and p65 is able to activate hTERT and also inhibit PINX1, leading to cell immortalization [60]. Taking this body of evidence into account, investigating the association between NF-κB p65 and viral LMP1 in RCC patients seems of great importance to confirm this potential mechanism through which EBV may play its role in the pathogenesis of RCC.

## Conclusions

In summary, the new findings of this study imply that EBV plays a part in RCC pathogenesis through the activation of NF-κB p65 signaling pathway, leading to the acceleration of tumor formation. However, it is noteworthy that the experimental results of this study may not adequately support the conclusion and more experiments such as examining the expression of viral RNA or EBV proteins need to be performed to draw more decisive conclusions.

## Data Availability

All data generated or analyzed during this study are available on reasonable request from the corresponding author.

## Abbreviations

RCC: Renal cell carcinoma
EBV: Epstein–Barr virus
EBNA2: EBV nuclear antigen 2
ccRCC: Clear cell RCC
Rb: Retinoblastoma protein
NF-κB: Nuclear factor-kappa B
IκB: NF-κB inhibitor
H&E: Hematoxylin and eosin
AJCC: American Joint Committee on Cancer
UICC: Union for International Cancer Control
VEGF: Vascular endothelial growth factor
PI3K/AKT: Phosphoinositide-3-kinase/protein kinase B
MAPK: Mitogen-activated protein kinase
JAK/STAT: Janus kinase/signal transducer and activator of transcription
TGF-β: Transforming growth factor-β
IHC: Immunohistochemistry
PCR: Polymerase chain reaction
nPCR: Nested polymerase chain reaction
